# 1092. Management of Transplant Graft and Role of Immunosuppression in the Outcome of Covid-19 Infection in Solid Organ Transplant Recipients

**DOI:** 10.1093/ofid/ofac492.932

**Published:** 2022-12-15

**Authors:** Suhel A Sabunwala, Tuhina Cornelius, Sadaf Aslam, Sally Alrabaa, Ambuj Kumar

**Affiliations:** University of South Florida Morsani College of Medicine, Memphis, Tennessee; University of South Florida Morsani College of Medicine, Memphis, Tennessee; University of South Florida Morsani College of Medicine, Memphis, Tennessee; Tampa General Hospital, Tampa, Florida; Morsani College of Medicine, University of South Florida, Tampa, Florida

## Abstract

**Background:**

COVID-19 disease became a global health care crisis and was declared pandemic by WHO in March 2020. Little is known how the immunosuppressive medications impact the mortality rate in Solid Organ Transplant (SOT) recipients. There is also minimal data regarding the incidence of transplanted graft failure or rejection that could be attributed to the COVID-19 infection itself or its complications and management. Our study aims to investigate the management of COVID-19 infection, outcome of the infection, transplant failure and rejection rates in SOT recipients.

**Methods:**

We conducted a retrospective cohort study of all consecutive SOT recipients who were admitted to our transplant center from March 2020 to April 2021 with COVID-19 infection. Data was collected from the electronic medical records after receiving Institutional Review Board approval.

**Table 1:**
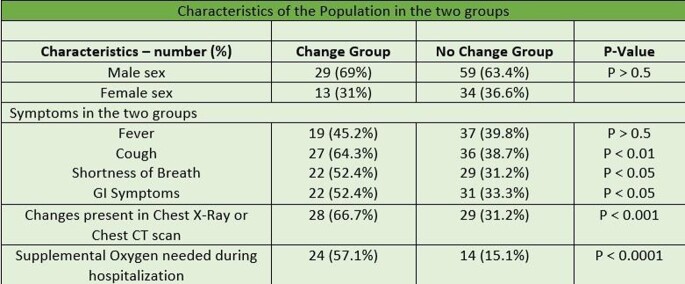
Characteristics of the Population in the two groups

**Table 2:**
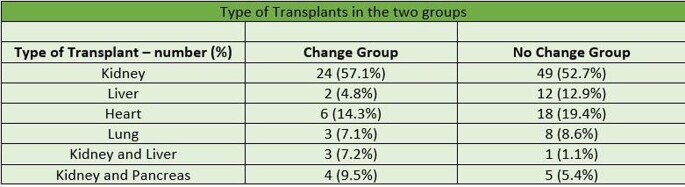
Type of Transplants in the two groups

**Results:**

A total of 135 patients met the inclusion criteria. After the diagnosis of COVID -19 infection, 31% recipients had decrease in the dose of immunosuppressive medications (change group) and 69% had no changes in the dose (no change group). Out of the 73 Kidney Transplant recipients 33% were in the change group compared to 14% of liver, 25% of heart and 27% of lung transplant recipients. Of the total 42 recipients in the change group, 28.6% required Intensive Care Unit (ICU) level care significantly higher compared to 7.5% in the no change group (p-value < 0.005). Mechanical ventilation was required in 14.3% of the patients in the change group and 6.5% in the no change group (p-value < 0.5). Out of the total, 85.7% patients in the change group survived compared to 94.6% in the no change group (p-value < 0.1). Overall, the transplant rejection rate was higher in the change group compared to the no change group (p-value < 0.5).

**Table 3:**
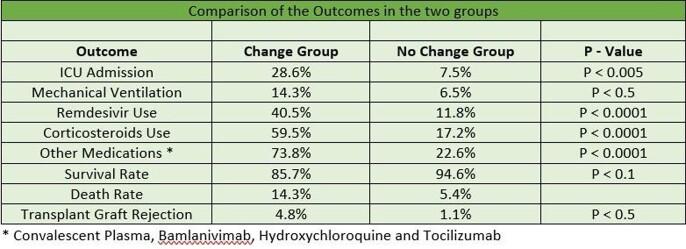
Comparison of the Outcomes in the two groups

**Figure 1:**
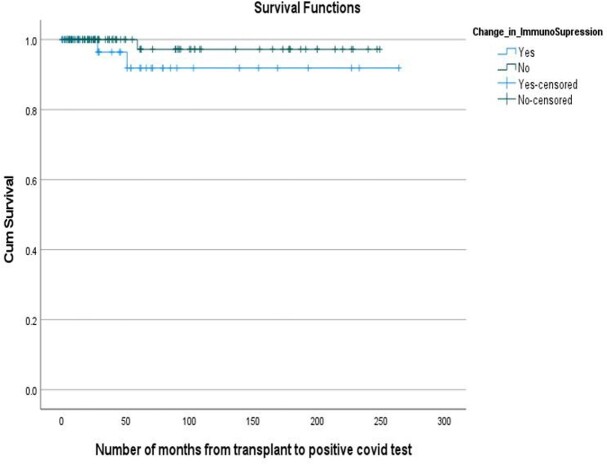
Kaplan – Meier Curve for survival

**Figure 2:**
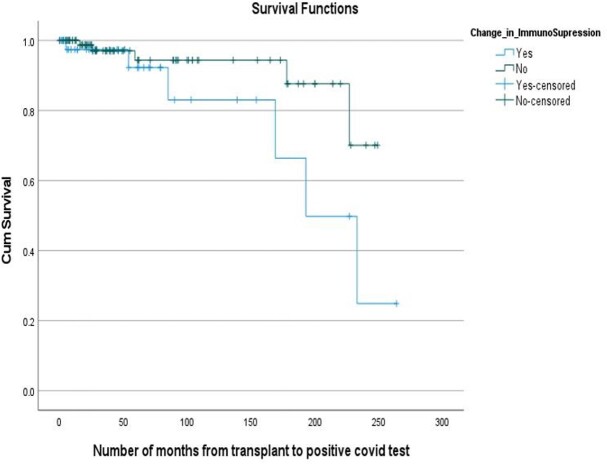
Kaplan – Meier Curve with Cox Regression for survival

**Conclusion:**

Our study showed a significantly higher ICU admission rate and mortality in SOT recipients who had their immune suppression reduced at the time of COVID-19 diagnosis. The same group also had a higher risk of rejection of transplanted graft. More studies with larger sample size needs to be done to further understand the management of immunosuppressive drugs in the SOT recipients with COIVD-19 infection.

**Disclosures:**

**All Authors**: No reported disclosures.

